# The “e-Generation”: The Technological Usage and Experiences of Medical Students from a Developing Country

**DOI:** 10.1155/2017/6928938

**Published:** 2017-08-24

**Authors:** Priyasdarshani Galappatthy, Wasundara S. Wathurapatha, Priyanga Ranasinghe, Maheshi D. M. S. Wijayabandara, Dinuka S. Warapitiya, Krishantha Weerasuriya

**Affiliations:** Department of Pharmacology, Faculty of Medicine, University of Colombo, Colombo, Sri Lanka

## Abstract

**Background:**

The medical community is increasingly using Portable Electronic Devices (PEDs). We evaluated usage of PEDs and medical apps among medical students from Sri Lanka.

**Methods:**

This descriptive cross-sectional study was conducted at Faculty of Medicine, University of Colombo. Medical students from 2nd to 5th year were invited for the study. A self-administered questionnaire was used to collect details of PEDs availability, accessibility, and usage, perceived advantages/barriers of PEDs, and availability, accessibility, and usage of medical apps.

**Results:**

Sample size was 505 (response rate, 61.8%). Mean age was 23.2 ± 1.3 years and majority were females (60.4%, *n* = 305). Majority (87.5%, *n* = 442) of students owned a PED. Nonaffordability was the most common reason for having not owning a PED (46%). Nonaffordability and lack of knowledge were key determinants of the usage of PEDs and medical “apps.” Doubts about reliability and lack of knowledge regarding reliable electronic sources of information were other significant barriers.

**Conclusions:**

Our results show that a significant majority of students owned a PED, a higher percentage than what is reported elsewhere. Considering barriers identified, it is important for institutions to promote usage of PEDs and medical apps by providing financial support, training, and knowledge to build confidence in technology.

## 1. Introduction

Portable Electronic Devices (PEDs), also known as hand-held digital devices, have rapidly moved from being considered a luxury to an essential part of human lives within a short period of time. Their ever-improving range of communication facilities, speed of processing, memory, and user-friendliness are all offered today at an affordable cost. Examples of PEDs include laptop computers, hand-held smart phones, tablet computers, media players, e-readers, and personal digital assistants. The introduction of smart phones and tablets, incorporating the use of numerous applications (“apps”) that influence day-to-day life, has been the most recent advance of this technology. The field of medicine and medical education has been no exception to this change. The use of Information and Communication Technology (ICT) is increasingly being recognized as an important part of medical education [1]. There is now an established repertoire of ICT implementations in medical education, including the use of online course materials, course management systems, and educational multimedia [2]. The medical community is increasingly using PEDs to access these services and resources. According to a recent systematic review, there is clear evidence of an increasing trend in PED use by health care professionals, especially in developed countries of the European region [3]. A recent multicentre survey done in UK showed that almost 99% of doctors owned and used a smart phone [4]. Furthermore, in developed countries smart phone ownership among medical students is known to be around 79–83% [5, 6].

The PEDs now provide easy access to medical journals, e-books, and websites and offer a wide range of medical apps for diagnostic, management, and drug reference purposes. This enables the so-called “e-generation” of medical students to “learn anywhere, anytime” with the ability to combine up to date data from multiple locations and organize content by themselves. The instant access to information while in the clinical setting gives the students a better framework for understanding and storing the new information and allows more efficient retrieval for future use [7]. Recent studies have shown that the “smart” use of such electronic devices can have a positive influence on the academic performance of medical students [8]. Quick and easy access to reliable and relevant evidence on a PED can improve learning in evidence based medicine and students' confidence in clinical decision making [9]. A 2012 systematic review, which examined published research on the use of the smart phones in the field of medicine, patient care, and continuing education, was able to find sixty research articles published up until May 2012 [10]. Despite the substantial number of studies, the authors concluded that there were only very few good-quality studies to answer many questions regarding the application and value of smart phones in medical education [10]. They highlighted the importance of conducting further research studies to evaluate how smart phones are being used.

Sri Lanka is a developing country in the South Asian region, with a population of nearly 21 million. The face of this electronic revolution has been quite strong in Sri Lanka, with nearly 25 million mobile subscribers (nearly 120% of the entire population) being registered by June 2016 [11]. However, at present the data regarding the use of PEDs among Sri Lankan students is lacking. The objective of the present study was to evaluate the usage of PEDs among medical students from Sri Lanka and document their technological experiences, perceptions, and attitudes toward technology and perceived barriers. We investigated the regularity with which medical students accessed and used technologies and technology-based tools and sought to determine the technologies and tools that were more and less favoured by medical students. We also explored the differences between preclinical and clinical students with regard to the usage of PEDs and technological experiences.

## 2. Materials and Methods

### 2.1. Study Population and Sampling

This descriptive cross-sectional study was conducted at the Faculty of Medicine, University of Colombo, Sri Lanka, in 2014. Established in 1870, the Faculty of Medicine, University of Colombo, is the second oldest medical school in South Asia. The undergraduate curriculum at the faculty spans 5 years and is conducted entirely in English. The initial 1.5 years of preclinical training focus on teaching the basic sciences of anatomy, physiology, and biochemistry (Introductory Basic Sciences Stream, IBSS). The student who completes this starts their clinical training in various specialties, with parallel academic teaching under a system based modular scheme (Applied Sciences Stream, ApSS) up until the end of their fourth year. In the final (fifth) year, teaching is mainly centered on clinical teaching under the five main specialties (clinical medicine, obstetrics and gynaecology, paediatrics, psychological medicine, and surgery). For the present study all students in their second to fifth (final) year of the faculty were invited. All students in the above-mentioned batches were invited and no predetermined sample size or sampling method was used. Informed written consent was obtained from all the participants, after explaining the purpose of the study and clarifying any queries. The participants were informed that they could refrain from or withdraw from filling the questionnaire at any point even after giving consent. Ethics approval for the study was obtained from the Ethics Review Committee, Faculty of Medicine, University of Colombo, Sri Lanka.

### 2.2. Study Instrument, Data Collection, and Analysis

An expert-validated pretested self-administered questionnaire in English, consisting of 5 subsections was used for data collection (see Supplementary File 1 in Supplementary Material available online at https://doi.org/10.1155/2017/6928938). Section one evaluated sociodemographic data, including age, gender, area of residence, mother's/father's occupation, and monthly family income. Section two and three looked at the details of PEDs availability, accessibility, and usage. Perceived advantages of PEDs in medical education and barriers to using PEDs were evaluated under sections four and five, while section six looked at the availability, the accessibility, and the usage of the internet among the students. The questionnaire was pretested among ten randomly selected students from the first year at the faculty, and ambiguous sections/questions were corrected, with the involvement of relevant experts from the fields of ICT and medical education where necessary.

Data were analyzed with Statistical Package for Social Sciences (SPSS) software, version 14. Descriptive data are presented as percentages or as mean ± SDs. Significance of associations was tested using Chi square for categorical variables and Student's *t*-test for continuous variables. In all analyses a *p* value ≤ 0.05 was considered statistically significant.

## 3. Results

### 3.1. Sociodemographic Characteristics

Sample size was 505 and the overall response rate was 61.8%. The response rates of the 2nd year, 3rd year, 4th year, and 5th year UGs were 36% (*n* = 79), 92.6% (*n* = 200), 50% (*n* = 96), and 65.6% (*n* = 130), respectively. Mean age (±SD) of study participants was 23.2 ± 1.3 years and the majority were females (60.4%, *n* = 305). The district of permanent residence of most UGs (30%, *n* = 151) was Colombo and most of UGs (42%, *n* = 212) had their secondary education from schools in the Colombo district. When parental employment status was considered among the study participants, 89.3% (*n* = 451) of fathers and 53.7% (*n* = 271) of mothers were employed. Monthly family income was in the range of LKR 10,000–50000 in the majority (56.5%, *n* = 285) (US$ ~ 65–330).

### 3.2. PEDs Availability, Accessibility, and Usage

The majority (87.5%, *n* = 442) of UGs owned a PED, while a further 6.9% (*n* = 35) of UGs have used a PED despite never owning one. Therefore, only a minority (4.8%, *n* = 24) have never used a PED. In those who did not own a PED, nonaffordability was the most common reason for having not owning a PED (46%). A minority of them did not like to use PEDs (8%) and were not aware of the additional advantages of PEDs over normal mobile phones (8%). The UGs who owned a PED more commonly used mobile smart phones (53%) than tablet PCs (15%) as their PED, while 30% of them used both mobile smart phones and tablets PCs. A significantly higher number of final year UGs (44%) were using both mobile smart phones and tablet PCs, while the majority of those from other junior batches were using only mobile smart phones (*p* < 0.05). The type of PED used was not associated with gender or monthly family income. However, monthly family income significantly predicted the respondent's ownership of a PED. All the UGs with a monthly family income > LKR 100,000 owned a PED, compared to 64% with income < LKR 10,000 (*p* < 0.05).

Final year UGs were significantly more likely (97%) to own a PED than their juniors (*p* < 0.05). There was no significant relationship between gender and the ownership of a PED (males: 87.6%, females: 88.8%). Most of the UGs have started using mobile smart phones (45.2%) and tablet PCs (81%) only after entering the university. However, most of respondents have used other (“nonsmart”) mobile phones (35%) and personal computers during school years (58%). Fifty-nine percent (*n* = 254) of UGs have started accessing medical information using PEDs before starting their clinical rotations, whereas 25% have started using PEDs only after starting their clinical appointments. Our results show that newer generations of UGs use PEDs to access medical information at an earlier time in their faculty life compared to older UGs (*p* < 0.05). For example, the majority (58%) of final year UGs have started accessing medical information using PEDs only after starting their clinical appointments, compared to majority of students in the junior batches (3rd year UGs, 72%, and 2nd year UG, 68%) who have started immediately after entering the Faculty. Almost all respondents (98.3%) used internet to access medical information. Mobile data connections (45.3%) were used more commonly than Wifi (35.9%) and cable connections (18.8%) to access the Internet. Gender or monthly family income was not significantly associated with Internet usage. Identified barriers to use of the internet were nonavailability of an internet connection (59.7%), nonavailability of a device to use the Internet (35.7%), and lack of knowledge regarding accessing and using the Internet (28.4%, *n* = 91).

### 3.3. Usage of Mobile Smart Devices to Access Medical Information

“Medscape” was the most commonly used mobile medical software (44.2%) by the UGs followed by the “BNF” (21.0%). The majority has accessed all these kinds of software via their mobile smart phones. Different sources of medical information (e-books, guidelines, journals, power point presentations, etc.) accessed by medical UGs via PEDs are shown in Table 1. The majority used PEDs to search for online information through the Internet (96%) and to go through power point presentations of lectures (82%). The three most commonly used e-books were Kumar & Clark's Clinical Medicine, British National Formulary, and Bailey & Love's Short Practice of Surgery. Sri Lanka National Guidelines were the most commonly accessed clinical guideline, followed by the NICE guidelines and RCOG guidelines. The most commonly accessed journals were The Sri Lanka Prescriber, New England Journal of Medicine (NEJM), and the Ceylon Medical Journal (CMJ). Considering mobile smart devices, tablet PCs were more commonly used than mobile smart phones to access all these sources by medical UGs.

Medical apps were most commonly used as an extra source of information related to academic content (30%), to find answers easily when answering questions (29%), to practice quizzes and tests which are available (21%), and to provide information to patients (20%). The majority (82%) of undergraduates believed medical apps are necessary, although most (71%) felt that medical apps were expensive. However, a considerable percentage (68%) thought that information available via medical apps was unreliable. Furthermore, 45% of respondents were not aware of how to gain access to these medical apps, which was more common among junior students (2nd year UG, 68%; 5th year UGs, 32%) and female students (53%) (*p* < 0.05). The percentage of UGs who believe that medical apps are a necessary accessory in their training significantly increased with seniority (2nd year UGs, 57%; 5th year UGs, 89%) (*p* < 0.05). In contrast to this, percentage of UGs thinking medical apps are unreliable and decreased with seniority (2nd year UGs, 90%; 5th year UG, 56%) (*p* < 0.05).

### 3.4. Perceived Advantages and Barriers to Use PED in Medical Education

Respondents perceived that quick access to medical information anywhere and anytime is the greatest benefit of PEDs (97.5%), followed by being convenient to use at the clinical settings (92%). Primary perceived barriers to this technology included unreliability of medical information (95%), fear of getting addicted to social networking (91%), and getting distracted from studies (84.3%). The student's source of encouragement to use PEDs to access medical information was fellow medical students (21%), lecturers at the faculty (19%), and clinicians during clinical training at the hospital (15%).

## 4. Discussion

To our knowledge, this is the first study from a South Asian country evaluating the PED availability and its usage to access medical information among medical UGs. Although portable knowledge appears to be the solution to cope with the overwhelming amount of continuously changing medical information, very little is known about how PEDs have been used in medical education and patient care, especially in developing countries like Sri Lanka. Our results show that 84.7% of UGs owned a PED. According to previous studies, 65% of Canadian and 38% of UK medical UGs owned hand-held computing devices. These lower rates are probably due to low response rate (14%) and sample sizes of the above studies and these studies being conducted 5-6 years ago, perhaps before the widespread use of PEDs [12, 13]. In a recent systematic review by Kho et al., 60–70% of medical students and residents used hand-held computers for educational purposes and patient care [14]. Hence, a higher percentage of Sri Lankan medical UGs own PEDs in comparison to what is reported in studies elsewhere. Similar to the present study, no significant association has been reported between gender and PED ownership in other studies [5, 8, 15]. But males were more likely to own and use medical “apps” [5] and were more likely to use PEDs in their clinical practice [16, 17]. One of the reasons for this could be that females are more likely to be unaware about how to gain access to medical “apps” as shown in the results of the present study.

Monthly family income significantly predicted the respondent's ownership of a PEDs and nonaffordability was the most common reason for not owning a PED in the present study population. A previous systematic review has also reported that cost is an important factor that affects the integration of hand-held devices into the medical setting and this includes the costs of medical software and support [18]. Another study among internal medicine residents demonstrated that cost of the equipment is a barrier to use hand-held computers [19]. Concern about the cost of PEDs and medical “apps” was an important finding even among students from developed countries, such as the UK [5]. Nevertheless, the costs of a hand-held device are significantly lower per unit than desktop or laptop computers [18]. Since the PEDs have numerous benefits in medical education compared to laptop or desktop computers, which cannot be used at the point of care for patients and during academic/teaching sessions, students and parents should be encouraged and facilitated to use PEDs. Government and various other institutions can provide financial support for medical UGs to purchase PEDs, thereby investing in increasing the quality of future health care providers. As reported by Safdari et al. financial support for purchasing smart phones and medical “apps” is the second most important factor in increasing use of this technology [20]. To implement these, further studies are required to evaluate the cost effectiveness of providing medical UGs with PEDs and its impact on improving quality of future health care.

As observed in the present study final year UGs were more likely (97%) to own PEDs. Similar findings have been observed in Canada where fourth year students had the highest rate (70.6%) of ownership of PEDs compared to juniors [3]. Grasso et al. also reported 28% of preclinical students and 76% of students in their clinical years used PEDs [21]. This is probably due to the fact that students use their PEDs mostly within the clinical context and less commonly during lecture sessions [13]. Medical trainees often use hand-held computers in clinical settings as portable resources providing rapid, point-of-care information to guide patient care and augment self-directed learning [14]. This explains the reason for the increased use of PEDs among final year UGs where teaching is mainly in clinical settings. “Medscape” was the most popular mobile software (44.2%) among our study participants, followed by the “BNF” (21%). “Prognosis,” “UpToDate,” and “Micromedex” software were rarely used by UGs in the present study. This is consistent with previous studies, which reported that most drug reference “apps” were the most frequently accessed medical apps by UGs [5, 12, 20–22]. However, in contrast, “UpToDate,” “Google,” “Medscape,” “Wikipedia,” and “Epocrates” were the most commonly used electronic resources by American UGs [23], whereas “Wikipedia” and “Pubmed” were the most commonly used by Indian UGs [24]. Availability of apps free of charge could account for the differences observed in different settings. In our study, the three most commonly used e-books were the Kumar & Clark's Clinical Medicine, BNF and Bailey & Love's Short Practice of Surgery. Davies et al. reported that the most popular e-books were BNF and Oxford Hand Book of Medicine [13]. These differences in usage of medical “apps” and e-books probably stem from the differences in their availability as well as differences in recommendations for text books provided by each university.

A considerable percentage (68%) of students in the present study thought that information available via medical “apps” was unreliable. Previous studies have also revealed concerns regarding reliability of information available via medical “apps” as challenges of PED usage [25, 26]. However, an interesting observation by Moore et al. showed that it is the majority of non-PED users who express concern about the reliability, security, and dependency of the PEDs and students who used PEDs perceived them to have greater value than nonusers [27]. This is also compatible with findings of the present study which showed an inverse relationship between increasing seniority and decreasing perception about reliability, as evidenced by increasing usage among senior UGs. Hence it is evident that doubts about reliability have become a significant barrier to the usage of PEDs among medical UGs. In order to overcome this barrier students should be taught of accurate and highly accessed medical resources. Health sciences libraries should be established to provide UGs the access to licensed, highly used, and accurate medical resources. This will help to improve confidence about reliability among UGs and provide breadth of resources, ease of access, and cost savings [22]. Furthermore, accreditation and quality assurance of medical “apps” by valid health institutions are other important ways to increase reliability and usage [20].

Another important barrier that we identified was that 45% of respondents were not aware of how to gain access to medical “apps,” more common among junior UGs. Chatterley and Chojecki reported that 35% of students regularly had problems in downloading programs, updating resources, and using wireless Internet and they were frustrated by lack of technical support [12]. Inexperience and lack of comfort with technology were also described as significant barriers in other studies [6, 25]. Safdari et al. showed that the technical skills of using medical “apps” of a significant percentage of medical UGs (31%) were only at an elementary level [20]. As a solution to this significant barrier, initiation of hand-held computer training in the first year of medical school with ongoing training during clinical rotations has been recommended [28]. Similarly other authors also recommended that mobile medical technology should be integrated at the beginning of UG medical education providing training and encouragement to medical students from the very first year [6, 29]. Therefore, institutions could promote PEDs usage by providing training and knowledge to build confidence in technology as a part of curriculum for medical UGs, especially during their early years.

Present study has several strengths, including being the first study from a South Asian country assessing the use of PEDs among medical UGs. Other strengths include larger sample size and involvement of four batches of UGs. Since this describes current usage trends and the types of resources most often utilized by medical UGs, libraries will be more able to make such resources available and provide training in using such resources. Failure to examine the relationship between PED use and academic performance of medical UGs is a limitation of the present study. Furthermore, the study was performed at a single medical faculty in Sri Lanka. Currently there are 8 medical faculties in Sri Lanka. Hence, our findings may be difficult to be generalized to other medical schools in Sri Lanka, where variability in socioeconomic background and availability of other resources may lead to different observations. The study instrument was expert-validated and pretested (face and content validity); however, we did not evaluate the construct validity (factor analysis), internal consistency, and reliability of the questionnaire.

## 5. Conclusions

Our results show that a significant majority of UGs owned a PED, a higher percentage than what is reported in studies elsewhere. Monthly family income significantly predicted the respondent's ownership of a PED. Nonaffordability and lack of knowledge were key determinants of the usage of PEDs and medical “apps.” Doubts about reliability and lack of knowledge regarding reliable electronic sources of information were other significant barriers. Therefore, institutions should promote PEDs usage by providing training and knowledge to build confidence in technology as a part of curriculum for medical UGs, especially during their early years.

## Supplementary Material

Data collection questionnaire.

## Figures and Tables

**Table 1:** Different electronic sources of medical information (e-books, guidelines, journals, power point presentations, etc.) used by medical UGs via PED.

Source of medical information	Frequency of use		
	*N* (%)		
	Never used	Occasionally used	Commonly used
e-books			
Kumar & Clark's Clinical Medicine	30 (12)	50 (19)	180 (69)
British National Formulary	34 (14)	39 (16)	174 (70)
Bailey & Love's Short Practice of Surgery	33 (15)	43 (19)	152 (66)
Davidson's Principles and Practice of Medicine	40 (18)	60 (27)	125 (55)
Clinical Pharmacology: Brown and Bennett	58 (27)	59 (27)	97 (46)
Illustrated Textbook of Pediatrics	72 (39)	37 (20)	74 (41)
Rang & Dale's Pharmacology	83 (44)	48 (25)	58 (31)
Nelson Textbook of Pediatrics	96 (61)	23 (15)	39 (24)

Clinical guidelines			
Sri Lanka National Guidelines	38 (22)	24 (14)	102 (64)
NICE guidelines	41 (25)	44 (26)	82 (49)
RCOG guidelines	62 (45)	14 (10)	63 (45)

Journals			
The Sri Lanka Prescriber	70 (69)	20 (20)	12 (11)
New England Journal of Medicine (NEJM)	57 (58)	23 (23)	18 (19)
Ceylon Medical Journal (CMJ)	62 (75)	14 (17)	6 (8)
British Medical Journal (BMJ)	54 (50)	34 (32)	21 (28)

Other			
Power point presentations of lectures	11 (4)	39 (14)	226 (82)
Online information through web search	8 (3)	7 (3)	254 (96)
