# Supplementary material for: The “e-Generation”: The Technological Usage and Experiences of Medical Students from a Developing Country
Source: Int J Telemed Appl. 2017 Aug 24;2017:6928938. doi: 10.1155/2017/6928938 (PMC5613644; doi:10.1155/2017/6928938)
Supplement: Supplementary file 1 — Data collection questionnaire. [file 6928938.f1.pdf]

Evaluation of usage of portable electronic devices to access information related academic and clinical work by medical students of Faculty of Medicine, University of Colombo, Sri Lanka.

DATA ENTRY SHEET

Serial number:…………………………………………

***Mobile smart devices** refer to **smart phones, tablets including I pads**

1. SECTION- DEMOGRAPHIC DATA

1.1. A/L batch: 2008 AL/ 2009 AL/ 2010 AL/ 2011 AL/ 2012 AL
1.2. Age:………………………………………………………...
1.3. Date of Birth:………………………………………………….
1.4. Sex: Male/ Female
1.5. District of permanent residence:………………………………
1.6. District of secondary education (grade 6-11):…………………………………………..
1.7. District of secondary education (grade 12-13):…………………………………………
1.8. Father's occupation:……………………………………………
1.9. Mother's occupation:…………………………………………..
1.10. Monthly family income:……………………………………...

2. SECTION- SMART PHONE USAGE

2.1. Have you ever owned a mobile smart device: Yes/ No
2.2. Despite not owning a mobile smart device, have you ever used a mobile smart device? Yes/ No (Specify from where………………………………………………)
2.3. What is the reason for having not owned a smart device ever before?
   I. Economically cannot afford to buy a device
   II. I do not like using mobile smart device
   III. I have never needed a mobile smart device
   IV. I am not aware about the additional advantages of a mobile smart device over a normal mobile phone.
   V. Other……………………………………………………………….

2.4. Are you currently using a mobile smart device for your personal use: Yes/ No

2.5. What is the type of mobile smart device/ computers you are using currently?

   I. Mobile smart phone (please specify the brand and the type…………………..)
   II. Tablet (including I pads) (please specify the brand and the type………………

Evaluation of usage of portable electronic devices to access information related academic and clinical work by medical students of Faculty of Medicine, University of Colombo, Sri Lanka.

2.6. When did you start using following devices personally for the first time in your life (Please tic the correct cage):

	Never used	Before grade 10	During grade 10-11	During grade 12-13	After completing ALs, but before attending university	After coming to the university
Any type of mobile phone						
Smart mobile phone						
Tablet (including I pads)						
Laptop/notebook						
Personal computer (PC)						
Other						

3. SECTION- EXTENT OF USAGE OF *MOBILE SMART DEVICES TO ACCESS MEDICAL INFORMATION

3.1.When did you start accessing medical information using a mobile smart device?

I. Before attending to the medical faculty
II. After attending to the medical faculty, but before start clinical appointments
III. After starting clinical appointments

Evaluation of usage of portable electronic devices to access information related academic and clinical work by medical students of Faculty of Medicine, University of Colombo, Sri Lanka.

3.2.Have you used the following to access the medical information and indicate the devices used for each

If you are using the given sources, please rank them on a scale of 0-5 (0 never, 5 very frequently within brackets ( ).<br><br>Please indicate the other examples you are using in the provided space.	Tick 1 or more devices, you use to access each category			
	Smart mobile phone	Tablet (including I pads)	Laptop/ notebook	Peronal computer (PC)
Ex:<br>XXXXXXXXXXX(xxxxxxx)<br>• xxxxxx (5)-offline/online/both<br>• xxxxxxx (1) – offline/online/both<br>• xxxxxxxxx (2) – offline/online/both<br>• *Another example* (4)-0ffline/online/both<br>• ……………….( )-offline/online/both	√		√	
Free mobile software (android, windows etc..)<br>• Medscape ( )-offline/online/both<br>• Prognosis ( )- offline/online/both<br>• Micromedex ( )-offline/online/both<br>• …………….( )-offline/online/both<br>• …………….( )-offline/online/both				
Installed unlicensed mobile software (android, windows etc…)<br>• UpToDate ( )-offline/online/both<br>• …………..( )-offline/online/both<br>• …………..( )-offline/online/both<br>• …………..( )-offline/online/both				
Purchased mobile software (android, windows etc…)<br>• UpToDate ( )-offline/online/both<br>• BNF ( )-offline/online/both<br>• …………..( )-offline/online/both<br>• …………..( )-offline/online/both				

If you are using the given sources, please rank them on a scale of 0-5 (0 never, 5 very frequently within brackets ( ).<br><br>Please indicate the other examples you are using in the provided space.	Tick 1 or more devices, you use to access each category			
	Smart mobile phone	Tablet (including I pads)	Laptop/ notebook	Personal Computer (PC)
E books<br>• Davidson ( )<br>• Kumar & Clerk ( )<br>• Bailey & Love ( )<br>• Rang & Dale ( )<br>• Bennett & Brown ( )<br>• BNF ( )<br>• Nelson ( )<br>• Illustrated paediatrics ( )<br>• Oxford hand books ( )<br>• Other …………………..( )<br>• Other …………………..( )<br>• Other …………………..( )				
Clinical guidelines<br>• National guidelines ( )<br>• NICE guidelines ( )<br>• RCOG guideline ( )<br>• Other …………………….( )<br>• Other …………………….( )<br>• Other …………………….( )				
Journals<br>• Prescriber journal ( )<br>• NEJM ( )<br>• BMJ ( )<br>• CMJ ( )<br>• Other …………………….( )<br>• Other …………………….( )<br>• Other …………………….( )				
Documents made by yourself (Ex: short notes as word documents) ( )				

Power point presentations of lecturers ( )				
Recordings of lectures/ teaching sessions( )				
Online information through web search (ex:google) ( )				

Evaluation of usage of portable electronic devices to access information related academic and clinical work by medical students of Faculty of Medicine, University of Colombo, Sri Lanka.

3.3. What are your attitudes towards the use of medical apps (Ex: MedScape) in your mobile smart device in a scale of 1-5

(Rate on a scale 0-5; 0-never,5-always)

I. I feel as medical apps are unnecessary
II. Unsure how to obtain medical apps
III. Medical apps are too expensive
IV. Do not trust the apps' content
V. Had unfavourable experiences with medical apps
VI. Other………………………………………………

3.4. Where do you use mobile smart device to access medical information?

(Rate on a scale 0-5; 0-never, 5always)

I. At home ( )
II. At faculty ( )
III. At hospitals during clinical appointments ( )
IV. During travelling ( )
V. Hostel/ Boarding place ( )
VI. During academic sessions ( )
VII. Other…………………………………( )

3.5. Rate the usage of following sources to access medical information.

(Rate on a scale 0-5; 0-never, 5always)

I. Printed books (which I own/ received from friends) ( )
II. Library ( )
III. Personal computer at home/ hostel ( )
IV. Laptop/ note book ( )

V. Computers at faculty (   )
VI. My *Mobile smart devices (smart phone/ tablet) (   )
VII. Other persons’ smart mobile devices (   )

3.6. For which purposes do you use applications in mobile smart devices?

(Rate on a scale 0-5; 0-never, 5always)

I. As an extra source of information related to the academic content (lectures, ward classes) (   )
II. To find answers easily when answering questions (practicing past papers) (   )
III. To practice doing quizzes and tests which are available as smart phone applications (   )
IV. When providing information to the patients (   )
V. Other…………………………………………………………(   )

3.7. From where do you get encouragement and support to use mobile smart device to access medical information?

(Rate on a scale 0-5; 0-never, 5always)

I. Lecturers (   )
II. Clinicians (   )
III. Para-medical staff (   )
IV. Colleague medical students (   )
V. Senior medical students (   )
VI. Friends in non-medical fields (   )
VII. Relations (   )
VIII. Other……………………………………..(   )

Evaluation of usage of portable electronic devices to access information related academic and clinical work by medical students of Faculty of Medicine, University of Colombo, Sri Lanka.

4. SECTION- PERCEIVED ADVANTAGE OF USING *MOBILE SMART DEVICES TO ACCESS MEDICAL INFORMATION

4.1. What are the advantages of using *Mobile smart devices to access medical information as you feel?

(Rate on a scale 0-5; 0 Strongly disagree, 5 Strongly agree)

I. Have a quick access to the medical information at anytime ( )
II. Have a quick access to the medical information at anywhere ( )
III. Contain reliable information ( )
IV. Convenient to use in clinical settings ( )
V. Other……………………………………………………………………………………
………………………………………………………………………………………….

5. PERCIEVED BARRIERS OF USING *MOBILE SMART DEVICES TO ACCESS MEDICAL INFORMATION

5.1. What are the barriers for using *Mobile smart devices to access medical information as you feel?

(Rate on a scale 0-5; 0 Strongly disagree, 5 Strongly agree)

I. Distracts the medical students from the studies ( )
II. Causes wastage of time ( )
III. Makes medical students addicted to use social networks like face book ( )
IV. Contained information is unreliable ( )
V. Inconvenient to use in clinical setting ( )
VI. Other……………………………………………………………………………………
………………………………………………………………………………………….

6. INTERNET ACCESS AND USAGE

6.1. How frequently do you use internet? (Rate on a scale 0-5; 0 never, 5 always)

6.2.How do you access internet? (Rate on a scale 0-5; 0 never, 5 very frequently)

I. Wifi ( )
   1. Faculty ( )
   2. Hostel/ Boarding place ( )
   3. Home ( )
   4. Other……………………( )

II. Mobile data connection (pre paid/post paid)
   1. Dongle ( )
   2. Directly through the *mobile smart device ( )

III. Cable connections (Ex:SLT) ( )
   1. Home ( )
   2. Faculty ( )

6.3. What are the barriers for using internet to you?

(Rate on a scale 0-5; 0 strongly disagree, 5 always)

I. Unavailability of a device (smart mobile device/computer) to use internet ( )
II. Lack of availability of an internet connection ( )
III. Lack of knowledge regarding accessing and using internet ( )
IV. Other………………………………………………..( )
